# Synthesis and Biological Evaluation of a Novel Pentagastrin-Toxin Conjugate Designed for a Targeted Prodrug Mono-therapy of Cancer

**DOI:** 10.3390/ijms9050821

**Published:** 2008-05-20

**Authors:** Lutz F. Tietze, Olaf Panknin, Birgit Krewer, Felix Major, Ingrid Schuberth

**Affiliations:** Institute of Organic and Biomolecular Chemistry, Georg-August-University Göttingen, Tammannstraße 2, D-37077 Göttingen, Germany

**Keywords:** antibiotics, antitumor agents, pentagastrin, prodrug, prodrug monotherapy

## Abstract

A novel carbamate prodrug **2** containing a pentagastrin moiety was synthesized. **2** was designed as a detoxified analogue of the highly cytotoxic natural antibiotic duocarmycin SA (**1**) for the use in a targeted prodrug monotherapy of cancers expressing cholecystokinin (CCK-B)/gastrin receptors. The synthesis of prodrug **2** was performed using a palladium-catalyzed carbonylation of bromide **6**, followed by a radical cyclisation to give the pharmacophoric unit **10**, coupling of **10** to the DNA-binding subunit **15** and transformation of the resulting *seco*-drug **3b** into the carbamate **2** via addition of a pentagastrin moiety.

## 1. Introduction

**Scheme 1.**
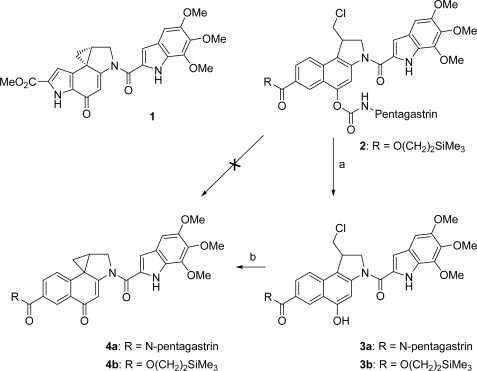
(+)-Duocarmycin SA (**1**), prodrug **2**, seco-drugs **3** and drugs **4**; a) enzymatic toxification of the novel carbamate prodrug **2** to the seco-drug **3b** inside the tumor cells and b) rapid cyclisation of the seco-drugs **3** *in situ* to give the cytotoxic drugs **4**.

One of the major problems in the chemotherapy of cancers is the usually low differentiation between normal and malignant cells by the known antiproliferating agents, resulting in severe side effects. Several approaches have been developed to overcome this problem like the antibody-directed enzyme prodrug therapy (ADEPT) [1,2] and the prodrug monotherapy (PMT) [3]. Whereas in ADEPT artificial antibody-enzyme conjugates are needed for targeting tumor cells, in PMT specific endogenous enzymes or receptors overexpressed in cancerous tissue are addressed to allow a selective killing of tumor cells. In both approaches, a relatively untoxic prodrug is used, which is then selectively converted into the corresponding cytotoxic drug in the cancer tissue; however, for PMT the prodrug is linked to a ligand which allows a targeting of cancer cells. Among these ligands small peptides play an important role having a low immunogenicity as well as high specifities and affinities to certain receptors which are overexpressed on certain tumour cells [4]. Some of these peptides that are already successfully applied in cancer therapy, belong to the gastrin family. For example, radiolabeled gastrin derivatives have shown a high therapeutic and diagnostic potential in targeting cholecystokinin (CCK-B)/gastrin receptor expressing tumors [5]. In addition, a gastrin derivative was linked to a triazene alkylating agent [6]. However, the observed receptor-mediated cytotoxicity of this conjugate was quite low. Better results were obtained with heptagastrin linked to an ellipticine derivative [7]. A high receptor-mediated cytotoxicity could be achieved with the anthracyclines daunorubicin, doxorubicin and 2-pyrrolinodoxorubicin as well as other cytotoxic agents like melphalan, cisplatin or methotrexate coupled to peptides of the LHRH [4,8], bombesin [4,8a,^9^], somatostatin [4,8a,^10^] and neuropeptide Y [4,11] type. Recently, we have developed the pentagastrin-toxin conjugate **3a** containing a *seco*-duocarmycin SA derivative (Scheme 1) [12].

Here, we report the synthesis of a novel pentagastrin conjugate **2** for the use in a targeted tumor therapy, which has the advantage over normal gastrin-toxin conjugates that a prodrug is used instead of a toxic drug (Scheme 1). In this concept, the pentagastrin moiety should serve not only as a targeting ligand for CCK-B/gastrin receptors, but also as a detoxifying unit. Thus, the corresponding drug **4b**, which again is an analogue of the naturally occuring antibiotic (+)-duocarmycin SA (**1**) with an IC_50_ value of 10 pm (L1210) [13], should be formed via **3b** inside the tumor cells by cleavage of the carbamate by lysosomal enzymes after endocytosis. The antiproliferative effect of **1** and its analogues such as the CBI-drugs **4** derive most probably from a selective alkylation of *N*-3 of adenine in DNA by nucleophilic attack at the spirocyclopropyl-cyclohexadienone moiety as the pharmacophoric group [14]. Since we have previously shown that the formation of a drug as **4b** from a *seco*-drug as **3b** is a very fast process and that the blocking of the phenolic hydroxyl group of **3b** allows a very strong reduction of its cytotoxicity, we used the *seco*-drug **3b** as substrate for the conjugation with the pentagastrin moiety, performing the connection to the phenolic hydroxyl group via a carbamate moiety [1b,1c,^15^].

**Figure 1.**
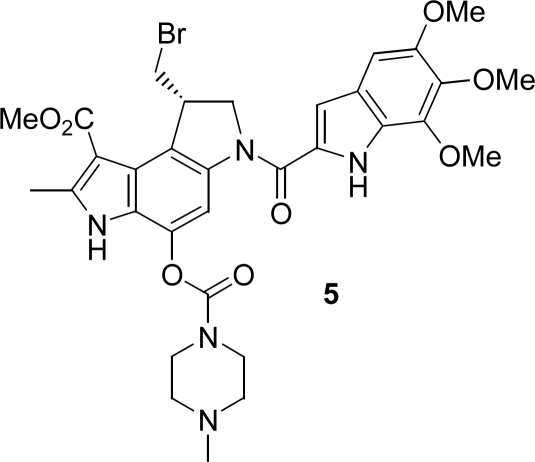
KW-2189 (**5**).

In our approach we did not employ the whole heptadecapeptide gastrin but the shorter *β*-alanine modified pentagastrin, because its *β*-Ala-Trp-Met-Asp-Phe-NH_2_ sequence representing the *C*-terminal amide of the natural peptides restores the biological activity of gastrin in a comparable order of magnitude [4e,^16^]. As a consequence, the *seco*-duocarmycin moiety **3b** had to be attached via the *N*-terminal amino functionality of pentagastrin using a carbamate. Such a carbamate substructure exists also in KW-2189 (**5**) (Figure 1) [17], an agent already investigated in clinical trials, and in several other anticancer agents [18].

For the formation of the carbamate moiety, we envisaged an addition of an isocyanate to the *seco*-drug **3b**. The TMSE ester moiety was introduced to allow a better comparison with the already prepared pentagastrin-conjugate **3a**. Moreover, the handle could be used for the introduction of a fluorescence dye to allow an investigation of the mode of action of such a compound employing a confocal laser scanning microscope.

## 2. Results and Discussion

### 2.1. Synthesis

**Scheme 2.**
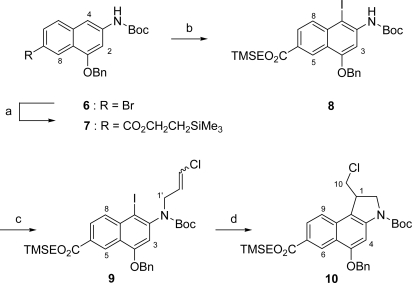
Synthesis of *seco*-CBI compound **10**. a) Mo(CO)_6_, 1 bar CO, 5 mol% Pd(PPh_3_)_2_Br_2_, 20 mol% dppf, *n*Bu_3_N, TMSEOH, DMF, 120 °C, 7 h, 56%; b) NIS, TsOH·H_2_O, THF/MeOH, 50 °C, 1 h, 73%; c) NaH, 1,3-dichloropropene, DMF, 20 °C, 13.5 h, 97%; d) HSi(SiMe_3_)_3_, AIBN, benzene, reflux, 2 h, 92%.

As starting material for the preparation of **2** we employed the known aminonaphthalin **6** [12]. **6** was converted into TMSE ester **7** in 56 % yield by a palladium-catalyzed carbonylation reaction using a CO atmosphere (1 bar) and Mo(CO)_6_ as additional CO source [19] in a mixture of 2-(trimethylsilyl)-ethanol and DMF (Scheme 2). The moderate yield of 56% of this carbonylation reaction might be due to the relatively high electron density of **6**. Nevertheless, **6** had to be used in the carbonylation reaction as the *Curtius* rearrangement of the corresponding acid to the protected naphtholamine could not be achieved after the introduction of the TMSE ester moiety. Iodination of **7** employing NIS [20,21] with TsOH·H_2_O as catalyst followed by *N*-alkylation of the formed **8** with 1,3-dichloropropene and subsequent radical cyclization [22] using the untoxic tris-(trimethylsilyl)-silan (TTMSS) [23] as hydride source and AIBN as radical starter provided *seco*-CBI derivative **10** in 65 % yield over three steps.

**Scheme 3.**
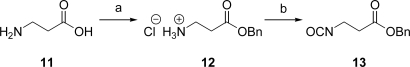
Synthesis of isocyanate **13**. a) TMSCl, BnOH, 20 °C, 15 h, 89%; b) triphosgene, toluene, reflux, 7.5 h, 98%.

In order to connect **10** with the peptide unit, we used the isocyanate **13** containing an ester moiety. This was first reacted with the phenolic hydroxyl group and then bound to the peptide via an amide linkage. The required isocyanate **13** was prepared as follows: first, *β*-alanine (**11**) was converted into the corresponding benzyl ester hydrochloride **12** employing TMSCl and benzylic alcohol [24]. Then, the isocyanate moiety was introduced using solid and thus easy to handle triphosgene in refluxing toluene to give **13** in 87 % yield over two steps (Scheme 3).

**Scheme 4.**
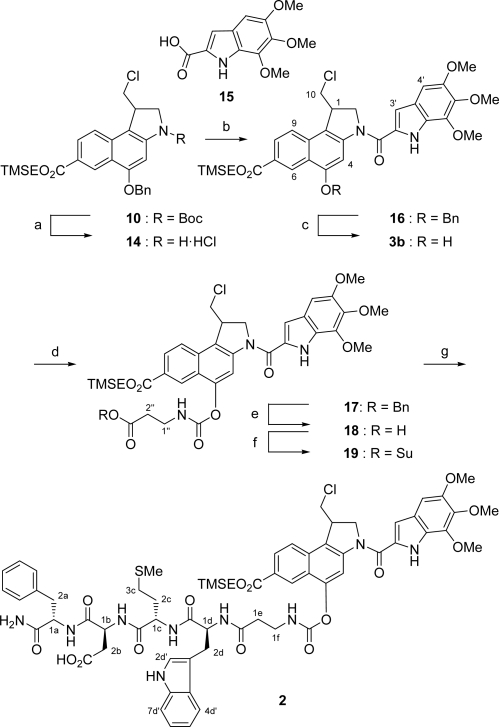
Synthesis of carbamate prodrug **2**. a) HCl/EtOAc, Et_3_SiH, CH_2_Cl_2_, 20 °C, 7 h; b) TMI-CO_2_H (**15**), EDC·HCl, DMF, 20 °C, 1 d, 43% (two steps); c) Pd/C, NH_4_HCO_2_, THF/MeOH, 20 °C, 75 min, quant.; d) isocyanate **13**, NEt_3_, CH_2_Cl_2_, 0–20 °C, 16 h, 83%; e) Pd/C, NH_4_HCO_2_, THF/MeOH, 20 °C, 25 min, 90%; f) HOSu, EDC·HCl, THF/CH_2_Cl_2_, 0–20 °C, 15 h; g) tetragastrin (**20**), NEt*i*Pr_2_, H_2_O/DMF, 20 °C, 7 h, 57% (two steps).

Hence, the synthesis of carbamate prodrug **2** was completed in seven further steps (Scheme 4). After deprotection of the secondary amino functionality in **10** under acidic conditions in an aqueous HCl/EtOAc mixture with Et_3_SiH as cation scavenger [25], the obtained hydrochloride salt **14** was directly coupled with the DNA-binding subunit TMI-CO_2_H (**15**) to give **16** in 43 % yield over two steps. Then, the benzyl ether moiety in **16** was cleaved by transfer hydrogenolysis with an aqueous ammonium formate solution and palladium on charcoal as the catalyst [26] to yield phenol **3b** which was subsequently coupled with isocyanate **13** to afford carbamate **17** in a very good yield of 83 % over two steps. The benzyl ester in **17** was cleaved again by using transfer hydrogenolytic conditions to give **18** in 90 % yield. This reaction had to be carefully monitored by TLC as the carbamate was sensitive to these conditions. Finally, carboxylic acid **18** was treated with HOSu/EDC·HCl and the resulting active ester **19** directly coupled with the fully unprotected tetrapeptide **20** [27] to yield carbamate prodrug **2** in 57 % (83 % based on recovered starting material) over two steps.

### 2.2. In vitro cytotoxicity tests

The *in vitro* cytotoxicity assays were carried out in duplicate with CCK-B/gastrin-receptor positive cells of the human pancreatic cell line MIA PaCa-2 and CCK-B/gastrin-receptor negative cells of the human bronchial carcinoma cell line A549 as control in six multiwell plates with concentrations of 10^2^, 10^3^ and 10^4^ cells per cavity. Incubation with various concentrations of the *seco*-drug **3b** and the prodrug **2** was performed in ultraculture medium (Table 1).

Prodrug **2** shows the same cytotoxicity as its corresponding *seco*-drug **3b** in the cell culture assays using the CCK-B/gastrin-receptor positive cell line (MIA PaCa) and the CCK-B/gastrin-receptor negative cell line (A549). Thus, the obtained IC_50_-values are almost identical in these four experiments. This indicates that prodrug **2** seems not to be stable under the used cell culture conditions. In fact, HPLC-MS-measurements revealed a decomposition of prodrug **2** under loss of the targeting pentagastrin moiety thereby forming the corresponding *seco*-drug **3b**.

We suppose that the unstability of the carbamate moiety can be traced back to the hydrogen atom at its nitrogen which in turn is part of the β-alanine moiety of pentagastrin. We therefore plan to replace the hydrogen by a carbon moiety though it is not known whether such a modification of the pentagastrin would interfere with the binding of the conjugate to the corresponding CCK-B/gastrin-receptor.

## 3. Experimental Section

General: All reactions were performed in flame dried glassware under an argon atmosphere. Solvents were dried and purified according to standard procedures and redistilled prior to use. TLC chromatography was performed on precoated aluminium silica gel SIL G/UV254 plates (Macherey-Nagel & Co.) and silica gel 60 (0.040-0.063 mm) (Merck) was used for column chromatography. IR: Bruker Vector 22. UV/VIS: Perkin-Elmer Lambda 2. ^1^H-NMR: Varian Mercury-200, Unity-300 (300 MHz), Unity Inova-600 (600 MHz). ^13^C-NMR: Varian Mercury-200 (50 MHz), Unity-300 (75 MHz), Unity Inova-600 (150 MHz). For ^1^H and ^13^C, CDCl_3_, [D_6_]DMSO and [D_7_]DMF were used as solvents. Chemical shifts are reported on a δ scale. Signals are quoted as s (singlet), d (doublet), t (triplet), q (quartet), m (multiplet), m_c_ (centered multiplet) and br (broad). MS: Finnigan MAT 95, TSQ 7000, LCQ. HRMS was performed using among others a modified peak matching technique, error ±2 ppm, with a resolution of ca. 10,000. Elemental analysis: Mikroanalytisches Labor des Institutes für Organische und Biomolekulare Chemie der Universität Göttingen.

**3-Amino-1-benzyloxy-*N*-(*tert*-butoxycarbonyl)-7-[2-(trimethylsilyl)-ethoxycarbonyl]-naphthalene (7):** A magnetically stirred and degassed solution of bromide **6** (3.60 g, 8.40 mmol), N(*n*Bu)_3_ (6.0 ml, 4.7 g, 25 mmol) and 2-(trimethylsilyl)-ethanol (6.0 ml, 5.0 g, 42 mmol) in DMF (35 ml) was treated with Pd(PPh_3_)_2_Br_2_ (332 mg, 420 μmol), 1,1'-bis-(diphenylphosphino)-ferrocene (931 mg, 1.68 mmol) and Mo(CO)_6_ (1.1 g, 4.2 mmol). The reaction mixture was degassed again, set under a carbon monoxide atmosphere (1 bar) and stirred for 7 h at 120 °C (preheated bath). The alcohol and tributylamine were distilled off under reduced pressure, the resulting red oil adsorbed on silica gel and subjected to column chromatography (pentane/EtOAc = 10:1 → 7:1) to afford ester **7** (2.32 g, 56 %) as orange solid. *R*_f_ = 0.43 (pentane/EtOAc = 7:1); ^1^H NMR (300 MHz, CDCl_3_): *δ* = 0.08 (s, 9 H, Si(CH_3_)_3_), 1.13–1.18 (m, 2 H, CH_2_SiMe_3_), 1.55 (s, 9 H, C(CH_3_)_3_), 4.43–4.49 (m, 2 H, CH_2_OC=O), 5.26 (s, 2 H, CH_2_Ph), 6.73 (brs, 1 H, NH), 7.08 (d, *J* = 1.5 Hz, 1 H, 2-H), 7.34–7.64 (m, 6 H, 5 × Ph-H, 4-H), 7.69 (d, *J* = 8.7 Hz, 1 H, 5-H), 8.02 (dd, *J* = 8.7, 2.1 Hz, 1 H, 6-H), 8.96 ppm (d, *J* = 2.1 Hz, 1 H, 8-H); ^13^C NMR (50 MHz, CDCl_3_): *δ* = –1.40 (Si(CH_3_)_3_), 17.35 (CH_2_SiMe_3_), 28.32 (C(CH_3_)_3_), 63.09 (CH_2_OC=O), 70.30 (CH_2_Ph), 80.97 (C(CH_3_)_3_), 99.48 (C-3), 106.31 (C-1), 121.59, 125.61 (C-4a, C-6), 125.31, 126.61, 126.87, 127.45, 128.04, 128.61 (C-5, C-7, C-8, 5 × Ph-C), 136.41, 137.13, 138.65 (C-2, C-8a, Ph-C*_i_*), 152.51, 156.23 (C-4, C=O), 167.11 ppm (C(O)OTMSE); MS (EI, 70 eV): *m/z* (%) = 493 (18) [M]^+^, 437 (10) [M – C_4_H_9_ + H]^+^, 91 (100) [C_7_H_7_]^+^.

**2-Amino-4-benzyloxy-*N*-(*tert*-butoxycarbonyl)-1-iodo-6-[2-(trimethylsilyl)-ethoxycarbonyl]-naphthalene (8):** A magnetically stirred solution of **7** (352 mg, 713 μmol) in 1:1 THF/MeOH (10 ml) was treated with a solution of TsOH·H_2_O (14 mg, 71 μmol) in THF (1 mL) and *N*-iodosuccinimide (322 mg, 1.43 mmol). The resulting mixture was warmed to 50 °C and stirred for 1 h. The reaction mixture was quenched with NaHCO_3_ (5 ml, saturated solution) and water and extracted with EtOAc (2 × 10 ml). The combined organic phases were washed with Na_2_S_2_O_3_ (1 × 15 ml, saturated solution) and brine (15 ml), dried (MgSO_4_) and concentrated under reduced pressure to give an orange oil. This material was adsorbed on silica gel and subjected to column chromatography (pentane/EtOAc = 20:1) to afford iodide **8** (320 mg, 73 %) as colorless foam. *R_f_* = 0.65 (pentane/EtOAc = 10:1); ^1^H NMR (300 MHz, CDCl_3_): *δ* = 0.08 (s, 9 H, Si(CH_3_)_3_), 1.13–1.18 (m, 2 H, CH_2_SiMe_3_), 1.59 (s, 9 H, C(CH_3_)_3_), 4.44–4.49 (m, 2 H, CH_2_OC=O), 5.31 (s, 2 H, CH_2_Ph), 7.33–7.60 (m, 5 H, 5 × Ph-H), 8.04 (d, *J* = 9.3 Hz, 1 H, 8-H), 8.09 (dd, *J* = 9.3, 1.8 Hz, 1 H, 7-H), 8.14 (s, 1 H, 3-H), 8.95 ppm (d, *J* = 1.8 Hz, 1 H, 5-H); ^13^C NMR (50 MHz, CDCl_3_): *δ* = –1.40 (Si(CH_3_)_3_), 17.34 (CH_2_SiMe_3_), 28.30 (C(CH_3_)_3_), 63.29 (CH_2_OC=O), 70.55 (CH_2_Ph), 79.16 (C(CH_3_)_3_), 81.53 (C-1), 100.13 (C-3), 122.77, 126.27, 136.16, 137.06 (C-4a, C-6, C-8a, Ph-C*_i_*), 125.62, 127.90, 128.07, 128.16, 128.58, 131.39 (C-5, C-7, C-8, 5 × Ph-C), 140.53 (C-2), 152.51, 156.60 (C-4, C=O), 166.66 ppm (C(O)OTMSE); MS (EI, 70 eV): *m/z* (%) = 619 (14) [M]^+^, 563 (10) [M – C_4_H_9_ + H]^+^, 535 (18) [M – C_4_H_8_ – CO]^+^, 491 (5) [M – I – H]^+^, 91 (100) [C_7_H_7_]^+^, 57 (36) [C_4_H_9_]^+^.

**(*E*/*Z*)-2-Amino-4-benzyloxy-*N*-(*tert*-butoxycarbonyl)-*N*-(3-chloro-2-propenyl)-1-iodo-6-[2-(trimethylsilyl)-ethoxycarbonyl]-naphthalene (9):** A magnetically stirred solution of 8 (54 mg, 87 μmol) in DMF (1.5 ml) was treated with NaH (5.20 mg, 60 % in oil, 218 μmol). Stirring was continued for 40 min at 20 °C before (*E*/*Z*)-1,3-dichloropropene (16.0 μl, 19.0 mg, 174 μmol) was added dropwise, and it was stirred for a further 13 h at 20 °C. The ensuing mixture was then adjusted to pH 5 with NH_4_Cl (saturated solution) and extracted with EtOAc (4 × 5 mL). The combined organic phases were washed with water (10 ml), brine (10 ml), dried (MgSO_4_) and concentrated under reduced pressure to give an orange oil. Subjection of this material to column chromatography (pentane/EtOAc = 10:1) gave iodide **9** (59 mg, 97 %) as pale yellow foam. *R*_f_ = 0.43, 0.52 (pentane/EtOAc = 10:1); ^1^H NMR (200 MHz, CDCl_3_): *δ* = –0.14/0.10 (2 × s, 9 H, Si(CH_3_)_3_), 1.14–1.22 (m, 2 H, CH_2_SiMe_3_) 1.30/1.58 (2 × s, 9 H, C(CH_3_)_3_), 3.78 (dd, *J* = 13.8, 6.4 Hz, 1 H, 1′-H_a_), 4.19–4.35 (m, 1 H, 1′-H_b_), 4.46–4.54 (m, 2 H, CH_2_OC=O), 5.31 (brs, 2 H, CH_2_Ph), 5.92–6.15 (m, 2 H, 2′-H, 3′-H), 6.65–6.85 (m, 1 H, 3-H), 7.30–7.55 (m, 5 H, 5 × Ph-H), 8.15 (d, *J* = 8.0 Hz, 1 H, 8-H), 8.25 (d, *J* = 8.0 Hz, 1 H, 7-H), 9.01–9.10 ppm (m, 1 H, 5-H); ^13^C NMR (50 MHz, CDCl_3_): *δ* = –1.40 (Si(CH_3_)_3_), 17.33 (CH_2_SiMe_3_), 28.19/28.46 (C(CH_3_)_3_), 45.76/48.97 (C-1′), 63.53/64.59 (CH_2_OC=O), 70.49/70.58 (CH_2_Ph), 80.88/81.39 (C(CH_3_)_3_), 94.52 (C-1), 107.93/108.51 (C-3), 120.87/121.96 (C-3′), 124.79, 128.26/128.29, 135.91/136.01, 137.51/137.64 (C-4a, C-6, C-8a, Ph-C*_i_*), 125.36, 127.00/127.20, 127.90/127.94, 128.17/128.21, 128.45, 128.72/128.76, 133.06 (C-5, C-7, C-8, C-2′, 5 × Ph-C), 144.60/144.98 (C-2), 153.37/153.59 (C-4), 155.99/156.12 (C=O), 166.40/166.43 ppm (C(O)OTMSE); MS (EI, 70 eV): *m/z* (%) = 694 (16) [M + H]^+^, 566 (18) [M – I]^+^, 510 (88) [M – I – C_4_H_9_ + H]^+^, 91 (100) [C_7_H_7_]^+^, 57 (16) [C_4_H_9_]^+^.

**(1*R*/*S*)-5-Benzyloxy-3-(*tert*-butoxycarbonyl)-1-chloromethyl-2,3-dihydro-1*H*-benz[*e*]indole-7-carboxylic acid [2-(trimethylsilyl)-ethyl] ester (10):** Through a magnetically stirred solution of iodide **9** (308 mg, 444 μmol) in benzene (13 ml) was bubbled argon for 45 min. The oxygen-free solution was then treated with tris-(trimethylsilyl)-silane (124 μl, 99.0 mg, 400 μmol) and AIBN (17.0 mg, 102 μmol) and stirred for 2 h under reflux. The ensuing mixture was adsorbed on silica gel and subjected to column chromatography (pentane/EtOAc = 10:1) to afford **10** (208 mg, 92 %) as pale yellow solid. *R*_f_ = 0.44 (pentane/EtOAc = 10:1); UV/VIS (CH_3_CN): *λ*_max_ (lg *ε*) = 217 (4.417), 271 (4.753), 354 nm (4.158); IR (KBr): ν̃ = 3388 (NH), 2955, 1700 (C=O), 1621, 1461, 1412, 1366, 1249, 1139, 928, 839 cm^–1^; ^1^H NMR (300 MHz, CDCl_3_): *δ* = 0.08 (s, 9 H, Si(CH_3_)_3_), 1.12–1.18 (m, 2 H, CH_2_SiMe_3_), 1.61 (s, 9 H, C(CH_3_)_3_), 3.45 (t, *J* = 10.5 Hz, 1 H, 10-H_b_), 3.88–4.02 (m, 2 H, 1-H, 10-H_a_), 4.14 (dd, *J* = 11.4, 9.0 Hz, 1 H, 2-H_b_), 4.27 (d, *J* = 11.4 Hz, 1 H, 2-H_a_), ), 4.44–4.49 (m, 2 H, CH_2_OC=O), 5.29 (s, 2 H, CH_2_Ph), 7.35–7.48 (m, 3 H, 3 × Ph-H), 7.54–7.59 (m, 2 H, 2 × Ph-H), 7.64 (d, *J* = 8.7 Hz, 1 H, 9-H), 7.87 (brs, 1 H, 4-H), 8.08 (dd, *J* = 8.7, 1.5 Hz, 1 H, 8-H), 9.02 ppm (d, *J* = 1.5 Hz, 1 H, 6-H); ^13^C NMR (125 MHz, CDCl_3_): *δ* = –1.41 (Si(CH_3_)_3_), 17.33 (CH_2_SiMe_3_), 28.41 (C(CH_3_)_3_), 41.38 (C-1), 46.36 (C-10), 53.11 (C-2), 63.14 (CH_2_OC=O), 70.42 (CH_2_Ph), 81.43 (C(CH_3_)_3_), 96.93 (C-4), 114.37, 121.50, 124.92, 132.37, 144.14 (C-3a, C-5a, C-7, C-9a, C-9b), 121.72 (C-9), 126.75 (C-6), 127.21 (C-8), 127.62, 128.07, 128.59 (5 × Ph-C), 136.34 (Ph-C*_i_*), 152.41 (C-5), 157.25 (C=O), 166.91 ppm (C(O)OTMSE); MS (EI, 70 eV): *m/z* (%) = 567 (4) [M]^+^, 511 (7) [M – C_4_H_9_ + H]^+^, 91 (90) [C_7_H_7_]^+^, 57 (30) [C_4_H_9_]^+^; HRMS: calcd for C_31_H_38_ClNO_5_Si: 567.2208; confirmed.

***β*-Alanine benzyl ester hydrochloride (12):** A magnetically stirred suspension of *β*-alanine (**11**) (1.00 g, 11.2 mmol) in benzylic alcohol (56.0 ml, 58.0 g, 539 mmol) was treated dropwise over a period of 10 min with trimethylsilylchloride (3.6 ml, 3.0 g, 28 mmol) and stirring continued for a further 15 h at 20 °C. The resulting clear solution was poured into Et_2_O (600 mL), the precipitate collected by filtration and washed with Et_2_O (100 mL). Drying of this material under reduced pressure gave hydrochloride **12** (2.15 g, 89 %) as white solid; ^1^H NMR (200 MHz, [D_6_]DMSO): *δ* = 2.79 (t, *J* = 7.0 Hz, 2 H, 2-H_2_), 3.03 (t, *J* = 7.0 Hz, 2 H, 3-H_2_), 5.13 (s, 2 H, CH_2_Ph), 7.32–7.41 (m, 5 H, 5 × Ph-H), 8.23 ppm (brs, 3 H, NH_3_^+^); ^13^C NMR (50 MHz, [D_6_]DMSO): *δ* = 31.34 (C-2), 34.47 (C-3), 65.87 (CH_2_Ph), 127.95 (2 × Ph-C), 128.01 (Ph-C*_p_*), 128.34 (2 × Ph-C), 135.73 (Ph-C*_i_*), 170.05 ppm (C=O); MS (ESI): *m/z* (%) = 180 (100) [M – Cl]^+^, 359 (100) [2M – Cl – HCl]^+^.

**3-Isocyano-propionic acid benzyl ester (13):** A magnetically stirred suspension of hydrochloride **12** (2.13 g, 9.88 mmol) in toluene (15 ml) was treated with triphosgene (2.93 g, 9.88 mmol) and heated to reflux for 7.5 h (end of HCl-evolution). The resulting solution was concentrated under reduced pressure to afford isocyanate **13** (1.99 g, 98 %) as yellow liquid which was used for the next reaction without further purification. *R*_f_ = 0.21 (pentane/EtOAc = 10:1); IR (film): ν̃ = 3349, 2957, 2277 (NCO), 1736 (C=O), 1498, 1176, 823, 752, 699 cm^–1^; ^1^H NMR (200 MHz, CDCl_3_): *δ* = 2.65 (t, *J* = 6.4 Hz, 2 H, 2-H_2_), 3.61 (t, *J* = 6.4 Hz, 2 H, 3-H_2_), 5.18 (s, 2 H, CH_2_Ph), 7.35–7.40 ppm (m, 5 H, 5 × Ph-H).

**(1*R*/*S*)-5-Benzyloxy-1-chloromethyl-3-(5,6,7-trimethoxyindole-2-carbonyl)-2,3-dihydro-1*H*-benz[*e*]indole-7-carboxylic acid [2-(trimethylsilyl)-ethyl] ester (16):** A solution of the protected amine **10** (369 mg, 650 μmol) in CH_2_Cl_2_ (5 ml) was treated with HCl (18 ml of a 4 m solution in EtOAc) and Et_3_SiH (105 μl, 76.0 mg, 650 μmol) and stirred for 7 h at 20 °C. The solvent was removed under reduced pressure and the ensuing residue treated with toluene (2 × 10 ml) and again concentrated under reduced pressure. The resulting crude hydrochloride **14** was dried under reduced pressure and then treated with 5,6,7-trimethoxyindole-2-carboxylic acid (**15**) (180 mg, 715 μmol), EDC·HCl (374 mg, 1.95 mmol) and DMF (16 ml) and stirred for 1 d at 20 °C. The reaction mixture was adjusted to pH 2 with HCl (2 n) and extracted with EtOAc (4 × 20 ml). The combined organic phases were washed with water (3 × 20 ml), brine (20 ml), dried (MgSO_4_) and concentrated under reduced pressure to give a brown solid. This material was adsorbed on silica gel and subjected to column chromatography (pentane/EtOAc = 3:1) to yield **16** (196 mg, 43 % over two steps) as green solid. *R*_f_ = 0.57 (pentane/ EtOAc = 2:1); UV/VIS (CH_3_CN): *λ*_max_ (lg *ε*) = 209 (4.758), 271 (4.470), 315 (4.465), 363 nm (4.524); IR (KBr): ν̃ = 3461 (NH), 2951, 1711 (C=O), 1624, 1527, 1459, 1408, 1309, 1107, 837, 747 cm^–1^; ^1^H NMR (300 MHz, CDCl_3_): *δ* = 0.09 (s, 9 H, Si(CH_3_)_3_), 1.13–1.19 (m, 2 H, CH_2_SiMe_3_), 3.42 (dd, *J* = 10.8, 10.2 Hz, 1 H, 10-H_b_), 3.90–4.05 (m, 11 H, 3 × OCH_3_, 1-H, 10-H_a_), 4.43–4.48 (m, 2 H, CH_2_OC=O), 4.56 (dd, *J* = 11.1, 8.7 Hz, 1 H, 2-H_b_), 4.71 (dd, *J* = 11.1, 1.8 Hz, 1 H, 2-H_a_), 5.25–5.31 (m, 2 H, CH_2_Ph), 6.85 (s, 1 H, 4′-H), 6.96 (d, *J* = 2.4 Hz, 1 H, 3′-H), 7.31–7.55 (m, 5 H, 5 × Ph-H), 7.62 (d, *J* = 9.0 Hz, 1 H, 9-H), 8.07 (dd, *J* = 9.0, 1.8 Hz, 1 H, 8-H), 8.21 (s, 1 H, 4-H), 9.03 (d, *J* = 1.8 Hz, 1 H, 6-H), 9.75 ppm (d, *J* = 2.4 Hz, 1 H, indole-NH); ^13^C NMR (75 MHz, CDCl_3_): *δ* = –1.47 (Si(CH_3_)_3_), 17.26 (CH_2_SiMe_3_), 42.72 (C-1), 45.88 (C-10), 55.11 (C-2), 56.11, 61.00, 61.36 (3 × OCH_3_), 63.18 (CH_2_OC=O), 70.30 (CH_2_Ph), 97.54 (C-4′), 98.87 (C-4), 106.75 (C-3′), 115.94, 123.45, 125.65, 131.71, 144.30 (C-3a, C-5a, C-7, C-9a, C-9b), 121.99 (C-9), 122.48, 125.61, 129.48, 138.72, 140.55 (C-2′, C-3a′, C-6′, C-7′, C-7a′), 126.47 (C-6), 127.12 (C-8), 127.44, 127.97, 128.49 (5 × Ph-C), 136.22 (Ph-C*_i_*), 150.08 (C-5′), 156.65 (C-5), 160.51 (C=O), 166.62 ppm (C(O)OTMSE); MS (ESI): *m/z* (%) = 723 (100) [M + Na]^+^, 1423 (75) [2M + Na]^+^, 699 (100) [M – H]^–^; HRMS: calcd for C_38_H_41_ClN_2_O_7_Si: 701.2444 [M + H]^+^; found: 701.2441.

**(1*R*/*S*)-1-Chloromethyl-5-hydroxy-3-(5,6,7-trimethoxyindole-2-carbonyl)-2,3-dihydro-1*H*-benz[*e*]indole-7-carboxylic acid [2-(trimethylsilyl)-ethyl] ester (3b):** A magnetically stirred solution of benzyl ether **16** (152 mg, 217 μmol) in 3:1 THF/MeOH (6 ml) was treated with 10 % Pd/C (62 mg) and dropwise with NH_4_HCO_2_ (572 μl of a 25 % solution in water, 2.26 mmol) and stirring continued for 75 min at 20 °C. The reaction mixture was filtered through a pad of Celite^®^ and it was thoroughly washed with MeOH and THF. The filtrate was dried (MgSO_4_) and concentrated under reduced pressure to give **3b** (133 mg, quant.) as yellow solid which was used for the next reaction without further purification. *R*_f_ = 0.25 (pentane/ EtOAc = 2:1); ^1^H NMR (300 MHz, [D_6_]DMSO): *δ* = 0.09 (s, 9 H, Si(CH_3_)_3_), 1.12–1.17 (m, 2 H, CH_2_SiMe_3_), 3.81–3.88 (m, 7 H, 2 × OCH_3_, 10-H_b_), 3.95 (s, 3 H, OCH_3_), 4.02 (dd, *J* = 11.1, 3.1 Hz, 1 H, 10-H_a_), 4.20 (m_c_, 1 H, 1-H), 4.41–4.50 (m, 3 H, CH_2_OC=O, 2-H_b_), 4.74 (dd, *J* = 11.1, 9.1 Hz, 1 H, 2-H_a_), 6.97 (s, 1 H, 4′-H), 7.07 (d, *J* = 2.0 Hz, 1 H, 3′-H), 7.91–7.98 (m, 3 H, 4-H, 8-H, 9-H), 8.82 (d, *J* = 1.0 Hz, 1 H, 6-H), 10.83 (s, 1 H, OH), 11.42 ppm (d, *J* = 2.0 Hz, 1 H, indole-NH); ^13^C NMR (75 MHz, [D_6_]DMSO): *δ* = –1.45 (Si(CH_3_)_3_), 16.87 (CH_2_SiMe_3_), 40.72 (C-1), 47.36 (C-10), 55.09 (C-2), 55.93, 60.84, 61.00 (3 × OCH_3_), 62.56 (CH_2_OC=O), 98.07 (C-4′), 100.82 (C-4), 106.31 (C-3′), 115.12, 123.06, 125.45, 131.92, 144.76 (C-3a, C-5a, C-7, C-9a, C-9b), 120.95, 123.12, 130.69, 139.00, 139.94 (C-2′, C-3a′, C-6′, C-7′, C-7a′), 123.94 (C-9), 125.89 (C-8), 126.02 (C-6), 149.19 (C-5′), 155.57 (C-5), 160.39 (C=O_TMI_), 165.89 ppm (C(O)OTMSE); MS (ESI): *m/z* (%) = 633 (100) [M + Na]^+^, 1243 (29) [2M + Na]^+^, 573 (100) [M – H – HCl]^–^, 609 (22) [M – H]^–^; HRMS: calcd for C_31_H_35_ClN_2_O_7_Si: 611.1975 [M + H]^+^; found: 611.1975.

**(1*R*/*S*)-5-(2-Benzyloxycarbonyl-ethylcarbamoyloxy)-1-chloromethyl-3-(5,6,7-trimethoxyindo-le-2-carbonyl)-2,3-dihydro-1*H*-benz[*e*]indole-7-carboxylic acid [2-(trimethylsilyl)-ethyl] ester (17):** A magnetically stirred solution of phenol **3b** (198 μmol, 121 mg) in CH_2_Cl_2_ (15 ml) at 0 °C was treated dropwise with **13** (203 μl, 203 mg; 990 μmol) and then triethylamine (139 μl, 100 mg, 990 μmol). The reaction mixture was warmed to 20 °C and stirred for a further 16 h. The ensuing solution was cooled to 0 °C, adjusted to pH 2 with HCl (2 n) and extracted with EtOAc (4 × 10 ml). The combined organic phases were washed with brine (10 ml), dried (MgSO_4_) and concentrated under reduced pressure to give a yellow solid. This material was adsorbed on silica gel and subjected to column chromatography (pentane/EtOAc = 1:1) to afford carbamate **17** (135 mg, 83 %) as yellow solid. *R*_f_ = 0.29 (pentane/EtOAc = 2:1); ^1^H NMR (300 MHz, [D_6_]DMSO, 100 °C): *δ* = 0.10 (s, 9 H, Si(CH_3_)_3_), 1.13–1.19 (m, 2 H, CH_2_SiMe_3_), 2.71 (t, *J* = 7.1 Hz, 2 H, 2″-H_2_), 3.47–3.53 (m, 2 H, 1″-H_2_), 3.85 (s, 6 H, 2 × OCH_3_), 3.98 (dd, *J* = 11.2, 6.8 Hz, 1 H, 10-H_b_), 4.00 (s, 3 H, OCH_3_), 4.09 (dd, *J* = 11.2, 3.4 Hz, 1 H, 10-H_a_), 4.39 (m_c_, 1 H, 1-H), 4.45–4.50 (m, 2 H, CH_2_OC=O), 4.58 (dd, *J* = 11.1, 2.6 Hz, 1 H, 2-H_b_), 4.80 (dd, *J* = 11.1, 9.2 Hz, 1 H, 2-H_a_), 5.17 (s, 2 H, CH_2_Ph), 6.99 (s, 1 H, 4′-H), 7.09 (d, *J* = 2.2 Hz, 1 H, 3′-H), 7.30–7.41 (m, 5 H, 5 × Ph-H), 8.04 (dd, *J* = 8.7, 1.4 Hz, 1 H, 8-H), 8.09 (d, *J* = 8.7 Hz, 1 H, 9-H), 8.24 (s, 1 H, 4-H), 8.62 (d, *J* = 1.4 Hz, 1 H, 6-H), 11.02 ppm (brs, 1 H, indole-NH); ^13^C NMR (75 MHz, [D_6_]DMSO, 100 °C): *δ* = –0.95 (Si(CH_3_)_3_), 17.61 (CH_2_SiMe_3_), 34.62/34.68 (C-2″), 37.58/37.60 (C-1″), 41.76 (C-1), 47.79/47.84 (C-10), 55.62 (C-2), 56.99 (OCH_3_), 61.38 (2 × OCH_3_), 63.36 (CH_2_OC=O), 66.15 (CH_2_Ph), 99.48 (C-4′), 107.05 (C-3′), 111.34 (C-4), 122.42, 124.17, 126.73, 132.19, 144.43 (C-3a, C-5a, C-7, C-9a, C-9b), 123.78, 126.36, 131.09, 139.56, 140.95 (C-2′, C-3a′, C-6′, C-7′, C-7a′), 124.29 (C-9), 125.41 (C-6), 126.78 (C-8), 128.23 (2 × Ph-C), 128.35 (Ph-C*_p_*), 128.81 (2 × Ph-C), 136.72 (Ph-C*_i_*), 149.15 (C-5′), 150.13 (OC(O)N), 156.16/1546.18 (C-5), 161.23 (C=O_TMI_), 166.19 (C(O)OTMSE), 171.23 ppm (C(O)OBn); MS (ESI): *m/z* (%) = 611 (100) [M – C(O)NH-*β*-Ala-OBn + H]^+^, 838 (18) [M + Na]^+^, 1653 (14) [2M + Na]^+^, 573 (100) [M – C(O)NH-*β*-Ala-OBn – HCl]^–^, 609 (55) [M – C(O)NH-*β*-Ala-OBn – H]^–^.

**(1*R*/*S*)-5-(2-Carboxy-ethylcarbamoyloxy)-1-chloromethyl-3-(5,6,7-trimethoxyindole-2-carbo-nyl)-2,3-dihydro-1*H*-benz[*e*]indole-7-carboxylic acid [2-(trimethylsilyl)-ethyl] ester (18):** A magnetically stirred solution of benzyl ester **17** (203 mg, 249 μmol) in 3:1 THF/MeOH (10 ml) was treated with 10 % Pd/C (83 mg) and dropwise with NH_4_HCO_2_ (653 μl of a 25 % solution in water, 2.59 mmol). Stirring was continued for 25 min (TLC-monitoring necessary as the alanyl residue is easily cleaved off) at 20 °C. The reaction mixture was filtered through a pad of Celite^®^, which was thoroughly washed with MeOH and CH_2_Cl_2_. The combined filtrates were concentrated under reduced pressure, the residue dissolved in CH_2_Cl_2_, dried (MgSO_4_) and concentrated under reduced pressure again to give a yellow oil. This material was adsorbed on silica gel and subjected to column chromatography (CH_2_Cl_2_/ MeOH = 20:1) to afford acid **18** (163 mg, 90 %) as pale yellow solid. *R*_f_ = 0.44 (CH_2_Cl_2_/MeOH = 10:1); ^1^H NMR (300 MHz, CDCl_3_): *δ* = 0.06 (s, 9 H, Si(CH_3_)_3_), 1.12 (m_c_, 2 H, CH_2_SiMe_3_), 2.74 (m_c_, 2 H, 2″-H_2_), 3.41 (m_c_, 1 H, 10-H_b_), 3.65 (m_c_, 2 H, 1″-H_2_), 3.83–4.07 (m, 11 H, 1-H, 10-H_a_, 3 × OCH_3_), 4.42 (m_c_, 2 H, CH_2_OC=O), 4.57–4.64 (m, 1 H, 2-H_b_), 4.71–4.74 (m, 1 H, 2-H_a_), 6.39 (brs, 1 H, NH), 6.87 (brs, 1 H, 4′-H), 6.99 (brs, 1 H, 3′-H), 7.62 (m_c_, 1 H, 9-H), 8.01 (m_c_, 1 H, 8-H), 8.41 (brs, 1 H, 4-H), 8.56 (brs, 1 H, 6-H), 9.99 ppm (brs, 1 H, indole-NH); ^13^C NMR (75 MHz, CDCl_3_): *δ* = –1.46 (Si(CH_3_)_3_), 17.34 (CH_2_SiMe_3_), 34.17 (C-2″), 36.99 (C-1″), 43.01 (C-1), 45.75 (C-10), 55.17 (C-2), 56.22, 61.31, 61.49 (3 × OCH_3_), 63.65 (CH_2_OC=O), 97.78 (C-4′), 107.11 (C-3′), 111.78 (C-4), 121.31, 124.21, 126.85, 131.42, 143.39 (C-3a, C-5a, C-7, C-9a, C-9b), 122.50 (C-9), 123.56, 125.86, 129.29, 138.68, 140.83 (C-2′, C-3a′, C-6′, C-7′, C-7a′), 125.93 (C-6), 126.52 (C-8), 149.00 (C-5′), 150.10 (OC(O)N), 154.39 (C-5), 160.58 (C=O_TMI_), 166.61 (C(O)OTMSE), 171.50 ppm (C(O)OH); MS (ESI): *m/z* (%) = 748 (90) [M + Na]^+^, 1473 (100) [2M + Na]^+^, 573 (96) [M – C(O)NH-*β*-Ala-OH – HCl]^–^, 1449 (100) [2M – H]^–^.

**(1*R*/*S*)-1-Chloromethyl-5-[2-(*N*-succinimidyloxycarbonyl)-ethyl-carbamoyloxy]-3-(5,6,7-tri-methoxyindole-2-carbonyl)-2,3-dihydro-1*H*-benz[*e*]indole-7-carboxylic acid [2-(trimethylsilyl)-ethyl] ester (19):** A magnetically stirred solution of acid **18** (110 mg, 152 μmol) and *N*-hydroxysuccinimide (26.0 mg, 228 μmol) in 1:1 THF/CH_2_Cl_2_ (10 ml) at 0 °C was treated with EDC·HCl (44.0 mg, 228 μmol). The reaction mixture was warmed to 20 °C and stirring continued for 15 h. The ensuing solution was then adjusted to pH 2 with HCl (2 n) and extracted with EtOAc (4 × 10 ml). The combined organic phases were washed with brine (10 ml), dried (MgSO_4_) and concentrated under reduced pressure to afford **19** as yellow solid which was used for the next reaction without further purification. *R*_f_ = 0.83 (CH_2_Cl_2_/MeOH = 10:1); MS (ESI): *m/z* (%) = 845 (90) [M + Na]^+^, 1667 (100) [2M + Na]^+^, 573 (100) [M – C(O)NH-*β*-Ala-OSu – HCl]^–^.

**(1*R*/*S*)-1-Chloromethyl-5-(l-phenylalaninamidyl-l-aspartyl-l-methionyl-l-tryptophyl-*β*-alanyl-carbonyloxy)-3-(5,6,7-trimethoxyindole-2-carbonyl)-2,3-dihydro-1*H*-benz[*e*]indole-7-carboxylic acid [2-(trimethylsilyl)-ethyl] ester (2):** A magnetically stirred solution of tetragastrin (**20**)^[27]^ (39.4 mg, 66.0 μmol) in water (1 ml) was treated with NEt*i*Pr_2_ (11.5 μl, 8.50 mg, 66.0 μmol) and dropwise with a solution of crude **19** (49 mg, 60 μmol) in DMF (3.5 ml). Stirring was continued for 7 h at 20 °C. The ensuing mixture was adjusted to pH 2 with HCl (2 n) and extracted with EtOAc (5 × 5 ml). The combined organic phases were washed with water (5 ml) and brine (5 ml), treated with toluene (5 ml) and concentrated under reduced pressure. The resulting yellow solid was adsorbed on silica gel and subjected to column chromatography (CH_2_Cl_2_/MeOH = 15:1 + 0.5 % HOAc → 10:1 + 0.5 % HOAc) to give **2** (45 mg, 57 % over two steps, 83 % based on recovery, 1:1 mixture of both diastereomeres) as light yellow solid. Further purification was achieved by HPLC. *R*_f_ = 0.27 (CH_2_Cl_2_/MeOH = 10:1 + 0.5 % HOAc); ^1^H NMR (600 MHz, [D_7_]DMF): *δ* = 0.10/0.12 (2 × s, 9 H, Si(CH_3_)_3_), 1.20 (m_c_, 2 H, CH_2_SiMe_3_), 1.98–2.07 (m, 5 H, 2c-H_2_, SCH_3_), 2.37–2.66 (m, 6 H, 2b-H_2_, 3c-H_2_, 1e-H_2_), 2.98–3.06 (m, 3 H, 2a-H_b_, 1f-H_2_), 3.17 (dd, *J* = 14.8, 8.4 Hz, 1 H, 2d-H_b_), 3.21–3.24 (m, 1 H, 2a-H_a_), 3.28–3.33 (m, 1 H, 2d-H_a_), 3.88 (s, 3 H, OCH_3_), 3.90 (s, 3 H, OCH_3_), 3.95 (dd, *J* = 11.1, 7.9 Hz, 1 H, 10-H_b_), 4.03 (s, 3 H, OCH_3_), 4.12 (dd, *J* = 11.1, 3.3 Hz, 1 H, 10-H_a_), 4.30 (m_c_, 1 H, 1-H), 4.42–4.47 (m, 1 H, 1c-H), 4.50 (m_c_, 2 H, CH_2_OC=O), 4.58 (m_c_, 1 H, 1a-H), 4.64 (m_c_, 1 H, 1b-H), 4.67 (dd, *J* = 10.7, 2.2 Hz, 1 H, 2-H_b_), 4.73 (m_c_, 1 H, 1d-H), 4.85 (dd, *J* = 10.7, 9.1 Hz, 1 H, 2-H_a_), 6.57 (s, 1 H, NH), 6.97 (m_c_, 1 H, 5d′-H), 7.05 (s, 1 H, 4′-H), 7.06 (m_c_, 1 H, 6d′-H), 7.17 (m_c_, 1 H, 1 × Ph-H), 7.19 (m_c_, 2 H, 3′-H, 2d′-H), 7.24–7.32 (m, 4 H, 4 × Ph-H), 7.37/7.39 (2 × d, *J* = 8.0 Hz, 1 H, 7d′-H), 7.57/7.63 (2 × d, *J* = 7.9 Hz, 1 H, 4d′-H), 7.73 (brs, 1 H, NH), 7.83 (brs, 1 H, NH), 8.02 (d, *J* = 8.8 Hz, 1 H, 9-H), 8.05 (dd, *J* = 8.8, 1.6 Hz, 1 H, 8-H), 8.11 (brs, 1 H, 4-H), 8.18–8.44 (m, 4 H, NH), 8.99 (d, *J* = 1.6 Hz, 1 H, 6-H), 10.82/10.87 (2 × s, 1 H, indole-NH_Trp_), 11.34 ppm (s, 1 H, indole-NH_TMI_); ^13^C NMR (150 MHz, [D_7_]DMF): *δ* = –1.46/–1.42 (Si(CH_3_)_3_), 15.01 (SCH_3_), 17.67/17.76 (CH_2_SiMe_3_), 28.12 (C-2d), 30.79 (C-3c), 31.88 (C-2c), 32.09 (C-1e), 35.02 (C-1f), 38.16 (C-2a), 39.40 (C-2b), 42.21 (C-1), 47.94 (C-10), 51.56 (C-1b), 53.65 (C-1c), 55.07 (C-1d), 55.27 (C-1a), 56.03 (C-2), 56.46/56.49, 61.36, 61.43 (3 × OCH_3_), 63.38 (CH_2_OC=O), 98.89 (C-4′), 101.95 (C-4), 107.19 (C-3′), 110.62 (C-3d′), 111.93 (C-7d′), 116.06, 124.35, 126.61, 131.78, 145.99 (C-3a, C-5a, C-7, C-9a, C-9b), 118.89 (C-5d′), 118.95 (C-4d′), 121.46/121.51 (C-6d′), 122.17, 129.86, 133.05, 139.97, 141.16 (C-2′, C-3a′, C-6′, C-7′, C-7a′), 123.75 (C-9), 124.41 (C-2d′), 126.85, 126.91 (C-6, C-8, Ph-C*_p_*), 128.51 (C-3ad′), 128.78 (2 × Ph-C), 129.83 (2 × Ph-C), 137.33 (C-7ad′), 139.04 (Ph-C*_i_*), 150.54 (C-5′), 152.52 (OC(O)N), 156.90 (C-5), 161.36 (C=O_TMI_), 162.20 (C=O_Ala_), 166.96 (C(O)OTMSE), 170.71, 171.25, 172.27, 172.33 (4 × C=O), 184.62 ppm (C(O)OH); MS (ESI): *m/z* (%) = 633 (100) [M – C(O)NH-pentapeptide + H + Na]^+^, 573 (100) [M – C(O)NH-pentapeptide – Cl]^–^; HRMS: calcd for C_64_H_74_ClN_9_O_15_SSi: 1304.4556 [M + H]+; found: 1304.4558; HPLC (preparative): column: Kromasil 100 C18; eluent: 85 % MeOH, 15 % H_2_O + 0.05 % TFA; flow: 12 mL/min; *R*_t_: 31–40 min.

**Cell culture**: Human bronchial carcinoma cells of line A549 (ATCC CCL 185) were kindly provided by the Institut für Zellbiologie, Universität Essen, and human pancreatic carcinoma cells Mia PaCa-2 by the Universitätsklinikum Göttingen, Abteilung Hämatologie und Onkologie. Cell lines were maintained as exponentially growing cultures at 37 °C and 7.5% CO_2_ in air in culture medium (DMEM (*Biochrom*) supplemented with 10 % fetal calf serum, 44 mm NaHCO_3_ (*Biochrom*) and 4 mm l-Glutamine (*Invitrogen*)).

***In vitro* cytotoxicity assays**: Cells of line A549 or MIA PaCa-2 were seeded in duplicates in 6 multiwell plates at concentrations of 10^2^, 10^3^ and 10^4^ cells per well. After cells were allowed to adhear, cells were washed in a serum-free incubation medium (Ultraculture medium, *Lonza*). Incubation with compounds **2** and **3b** was then performed in Ultraculture medium at various concentrations for 24 h. All substances were used as freshly prepared solutions in DMSO (*Merck*) diluted with incubation medium to a final concentration of DMSO of 1% in the wells. After exposure the test substance was removed and cells were washed with fresh medium. Cultivation in normal growth medium was done for 10 days. The medium was removed, the clones were dried and stained with Löffler's methylene blue (*Merck*) and then counted macroscopically.

The IC_50_ values are based on the relative colony-forming rate, which was determined according to the following formula: relative colony-forming rate [%] = 100 × (number of clones counted after exposure) / (number of clones counted in the control).

## Figures and Tables

**Table 1.** *In vitro* cytotoxicity of prodrug **2** and of seco-drug **3b** against CCK-B/gastrin-receptor positive cells of the human pancreatic cell line MIA PaCa-2 and CCK-B/gastrin-receptor negative human bronchial carcinoma cells (A549). Cells were exposed to various concentrations of the test substance for 24 h at 37 °C; after 10 days of incubation following the exposure to the substance, clone formation was compared to an untreated control assay and the relative colony-forming rate was determined. IC_50_ is the drug concentration required for 50% growth inhibition of target cells.

Compound	MIA PaCa-2 IC_50_ [nm]	A549 IC_50_ [nm]
**2**	0.31	0.11
**3b**	0.31	0.14
